# The Discovery of a *Citrus Yellow Vein Clearing Virus* Hacienda Heights Isolate Diversifies the Geological Origins of the Virus in California, United States

**DOI:** 10.3390/v16091479

**Published:** 2024-09-18

**Authors:** Yong-Duo Sun, Raymond Yokomi

**Affiliations:** United States Department of Agriculture, Agricultural Research Service, San Joaquin Valley Agricultural Sciences Center, Parlier, CA 93648, USA

**Keywords:** citrus yellow vein clearing virus, genome sequencing, phylogenetic reference, virus origin

## Abstract

The citrus yellow vein clearing virus (CYVCV) is an emerging threat to the U.S. citrus industry. Reports from China shows it cause significant reductions in fruit yield and growth, particularly in lemon trees. In 2022, CYVCV was detected in a wide range of citrus cultivars in localized urban properties in Tulare, California. In 2024, a CYVCV-infected lemon tree was detected in Hacienda Heights in Los Angeles County, California, geographically separated from the Tulare foci. Through long-read sequencing technology, the whole-genome sequence of a CYVCV isolate from Hacienda Heights (designated as CYVCV-CA-HH1, Accession number PP840891.1) was obtained. Sequence alignments and neighbornet analysis strongly suggested that the CYVCV-CA-HH1 isolate has a different origin than the Tulare CYVCV (CYVCV CA-TL) isolates. The CYVCV CA-TL isolates were grouped with those from South Asia (India and Pakistan) and the Middle East (Türkiye), while the CYVCV-CA-HH1 isolate was grouped with isolates from East Asia (China and South Korea). Maximum likelihood phylogenetic analysis further supports this finding, showing that the CYVCV-CA-HH1 isolate shares the most recent common ancestor with a South Korean lineage, which derives from Chinese isolates. Together, our data suggest a diverse geological origin of CYVCV isolates in California.

## 1. Introduction

The citrus yellow vein clearing virus (CYVCV) is a positive-sense flexuous RNA virus, belonging to the *Alphaflexiviridae* family, *Potexvirus* genus, and the *Mandarivirus* subgenus [1,2,3]. Disease symptoms and intensities of CYVCV vary significantly depending on virus strains, citrus varieties, and environmental conditions [4,5]. Lemon (*Citrus limon*) and sour orange (*C. aurantium*) trees are highly symptomatic, while many other citrus cultivars, though susceptible, typically remain asymptomatic but can show mild vein clearing and leaf distortion symptoms occasionally under favorable conditions. Generally, chronic symptoms in CYVCV-infected tree leaf include deformities, yellow clear veins on the adaxial side, water-soaked veins on the abaxial side, venial necrosis, and intermittent ringspots. The diseased tree can become stunted and suffer yield loss [3]. In China, the rapid spread of CYVCV has caused substantial losses in lemon production where disease incidence is high [6]. Unfortunately, no management strategies have been proved effective to control CYVCV.

The first report of yellow vein clearing disease was detected in 1988 in Pakistan [7]. Since then, it has been reported in various locales, including Türkiye, India, Iran, China, and South Korea [8,9,10,11,12]. In 2022, during a routine multi-pest survey conducted by the California Department of Food and Agriculture (CDFA), CYVCV-infected citrus trees were identified in urban properties in Tulare, California [13,14,15]. This marked the first appearance of the virus in the western hemisphere. Abrahamian et al. [15] sequenced 17 CYVCV isolates from samples collected in Tulare, CA via high-throughput Nanopore sequencing and traditional Sanger sequencing. Through whole-genome sequence alignment with other 52 CYVCV isolates, they claimed that the California isolates fall into two major clades, with one being a CYVCV California-Tulare phylogroup. Meanwhile, our group has obtained three CYVCV California-Tulare isolates by long-read sequencing. By comparing them with all available full-length CYVCV sequences ever reported, we found that all three isolates share a common ancestor with an Indian isolate, suggesting a possible origin of CYVCV American isolates from South Asia (India) [13,14].

Recently, a lemon tree in a residential property of Hacienda Heights, California, was found to be infected with CYVCV and exhibited typical disease symptoms. This marks the second region in California reporting the presence of CYVCV. Hacienda Heights is in southern California, while Tulare is in central California; the locations are geographically separated by the Tehachapi Mountain range (Supplementary Figure S1) and approximately 276 km. Although the virus is reported to be vector-borne, these detection sites appear to be independent introductions. Additionally, several new CYVCV isolates have been reported to Genbank, NCBI from other parts of the world [12]. Given these updates, there is merit in reconstructing the phylogenetic references and updating the possible origin of CYVCV California isolates. In this study, we obtained the genome sequence of a CYVCV California-Hacienda Heights isolate, designated as CYVCV-CA-HH1, through long-read genome sequencing. Genome alignment, neighbornet analysis, and maximum likelihood phylogenetic reference indicate that all CYVCV isolates found in Tulare, CA may derive from isolates in South Asia (India and Pakistan). In contrast, the CYVCV-CA-HH1 isolate was grouped with East Asian isolates and shares the most common ancestor with a South Korea lineage. Together, these data suggest a potential diverse geological origin of CYVCV America isolates.

## 2. Materials and Methods

### 2.1. Sample Collection and Virus Quantification

Citrus budwood and leaf samples, from a CYVCV-positive lemon tree located in Hacienda Heights, California, were collected in 2024 and propagated in a containment greenhouse under a permit from the CDFA on a variety of citrus cultivars, including sour orange, Eureka lemon, and Madam vinous. The grafted plants were maintained in an air-conditioned greenhouse at the San Joaquin Valley Agricultural Sciences Center in Parlier, California. Total RNA from the CYVCV isolates was extracted from symptom-exhibiting branches using Trizol Reagent (ThermoFisher Scientific, Waltham, MA, USA). A one-step real-time quantitative Polymerase Chain Reaction (RT-qPCR) was employed for absolute quantification of CYVCV. Briefly, a standard curve was generated using a CYVCV California isolate cDNA infectious clone (unpublished data). The RT-qPCR reaction was performed as previously described [14]. The primers and probes were reported in Abrahamian et al., 2024 [15]. RNA samples at a concentration of 10 ng/µL were tested in triplicate.

### 2.2. CYVCV CA-HH1 Isolate Genome Sequencing

Next, 5′ and 3′ Rapid Amplification of cDNA Ends (RACE) were applied to pinpoint the 5′ and 3′ end of CYVCV CA-HH1, respectively [13]. Shortly, a conserved region at the 5′ end, specified through alignment with other reported CYVCV isolates, acted as the basis for developing a virus-specific 5′ RACE primer (5′-GGTTAGTGGTATTGCCCTGTT-3′). For the 3′ RACE-specific primer, an oligo(dT) primer was operated. The amplicons yielded from the 5′ and 3′ RACE PCRs were purified and subsequently cloned into the pGEM-T Easy vector (Promega Corp., Madison, WI, USA). The corresponding vectors were subjected to Nanopore sequencing (Plasmidsaurus, Arcadia, CA, USA) to obtain the sequences of the CYVCV 5′ and 3′ termini.

Using these 5′ and 3′ termini sequences, the complete genome sequences were amplified for CYVCV CA isolates using the Q5 high-fidelity enzyme (New England Biolabs Inc., Ipswich, MA, USA) and virus-specific PCR primers (5′ primer: GAAAAGCAAACATAACCAACACACACCC; 3′ primer: CAGAAAATGGAAACTGAAAGCCTGAATATTT). This yielded a 7.5 Kb PCR amplicon which was sequenced with the latest long-read sequencing technology from Oxford Nanopore Technologies (ONT, Plasmidsaurus, Arcadia, CA, USA). The fully assembled genome sequences were annotated and deposited in GenBank under the accession number PP840891.1 (CYVCV-CA-HH1).

### 2.3. Nucleotide Diversity Analysis

To analyze nucleotide diversity, the complete genomes of 21 CYVCV California isolates were uploaded and aligned using the NCBI Multiple Sequence Alignment Viewer. The Virus Intergenomic Distance Calculator (VIRIDIC) was utilized to develop a heatmap employing default sets that comprised intergenomic similarity values and alignment indicators [16].

### 2.4. Recombination Analysis

Recombination analysis of 76 distinct CYVCV isolates was accomplished using the Recombination Detection Program v4.56 (RDP4) software [17]. The software employed various algorithms, including BOOTSCAN, CHIMAERA, GENECONV, MAXCHI, RDP, SISCAN, and 3SEQ, each identifying putative recombination possibilities, major and minor parents, and breakpoints. Recombination events detected by at least three different algorithms were considered.

### 2.5. Construction of Neighbornet and Maximum Likelihood Phylogenetic Tree

The complete genomes of 76 CYVCV isolates were acquired from GenBank (NCBI) and aligned using MEGA 11 with the MUSCLE algorithm. A neighbornet reference was assembled and modified using SplitsTree 4, with 1000 bootstrap replicates [18].

The complete genomes of 70 non-recombinant CYVCV isolates were acquired for maximum likelihood (ML) reference. A ML tree was estimated using a non-clock (unconstrained) generalized time-reversible (GTR) + gamma substitution model via IQ-Tree [19]. The ML midpoint-rooted tree was visualized and adjusted using FigTree v1.4.4.

## 3. Results and Discussion

**Figure 1:**
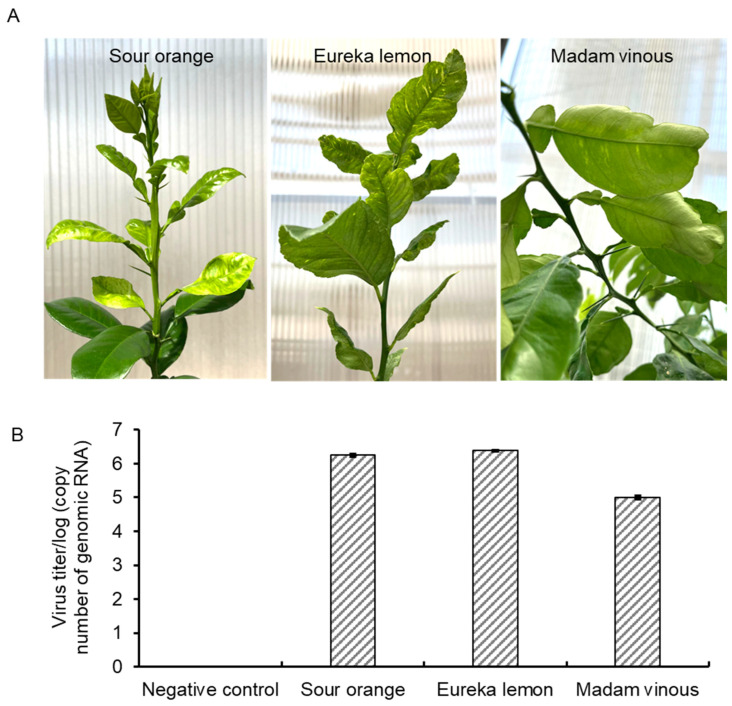
Disease symptoms triggered by a citrus yellow vein clearing virus (CYVCV) California-Hacienda Heights isolate in different citrus host. (**A**) A typical branch of Sour orange (*Citrus aurantium* L.), Eureka lemon (*Citrus limon*) and Madam vinous (*Citrus sinensis* (L.) Osbeck) plants exhibiting typical CYVCV-induced yellow vein clearing phenotype at three months post-graft inoculation in the greenhouse. (**B**) Absolute quantification of virus titers in the three hosts present in (**A**) by RT-qPCR. Three biological replicates yielded consistent results.

CYVCV was acquired from a lemon tree exhibiting CYVCV symptoms located in Hacienda Heights, CA, USA and propagated in the greenhouse. Propagations of three sour orange (*C. aurantium* L.) and Eureka lemon (*C. limon*) exhibited clear CYVCV symptoms of vein clearing, water soaking, and leaf distortion three months post inoculation. In contrast, Madam vinous (*Citrus sinensis* (L.) Osbeck) exhibited a relatively mild symptom of leaf chlorotic mottling and leaf crinkling (Figure 1A). The presence of CYVCV in the grafted plants was confirmed by RT-qPCR (Figure 1B). In alignment with the phenotype observation, the virus titer in the Madam vinous was lower compared to those in Sour orange and Eureka lemon.

The complete genome of the CYVCV isolate was sequenced through long-read sequencing and designated as CYVCV CA-HH1 (Accession number PP840891.1). The genome of CYVCV CA-HH1 consists of 7530 nucleotides (nt), excluding the 3′ poly-A tail, which is consistent with the 20 CYVCV isolates detected in Tulare, California. Multiple sequence alignment displayed base-pair differences with variations, indicating that CYVCV CA isolates exhibit relatively low divergence and limited heterogeneity (Supplementary Figure S2). Within this scope, the sequences of the CYVCV CA in-group isolates showed intergenomic similarities ranging from 96.9% to 99.9% (Supplementary Figure S3).

**Figure 2:**
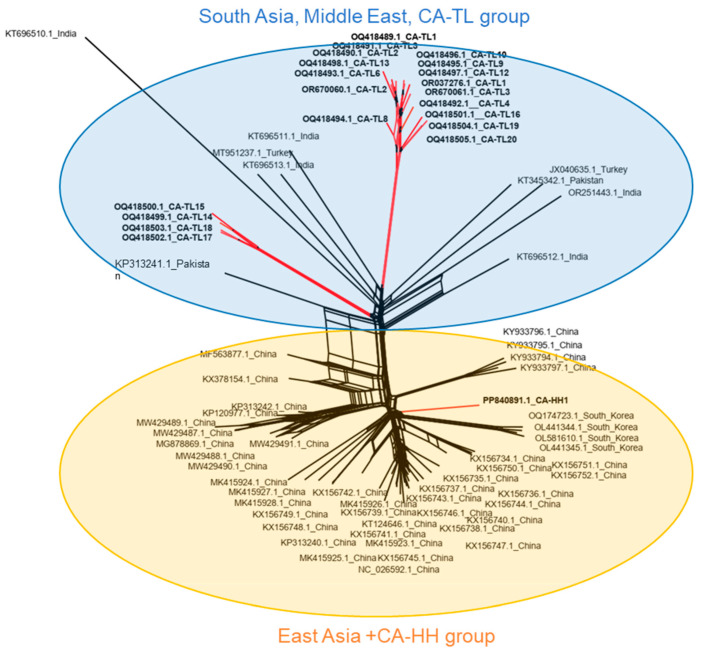
Construction of non-rooted Neighbornet phylogenetic tree upon 76 citrus yellow vein clearing virus (CYVCV) whole genome sequence. Briefly, CYVCV isolates could be divided into two major genotype groups. The isolates detected from South Asia (India and Pakistan), Middle East (Turkiye), and California-Tulare were grouped together (Light blue shade). The other isolates from East Asia (China and South Korea) and California-Hacienda Heights were grouped together (Light orange shade). The lineages of CYVCV CA isolates are marked red.

Sequence alignment of all 76 known CYVCV isolates (Supplementary Table S1) using a neighbornet reconstruction of CYVCV complete genomes unveiled two major genotype groups (Figure 2). All CYVCV isolates from China and South Korea formed a major group termed the “East Asia” group. Interestingly, the CYVCV HH isolate was categorized within the “East Asia” group, leading us to refer to this group as the “East Asia + CA-HH group”. The other CYVCV variants were grouped into another major genotype, termed the “South Asia, Middle East, CA-TL group,” which encompasses variants isolated from India, Pakistan, Türkiye, and Tulare, California (Figure 2).

**Figure 3:**
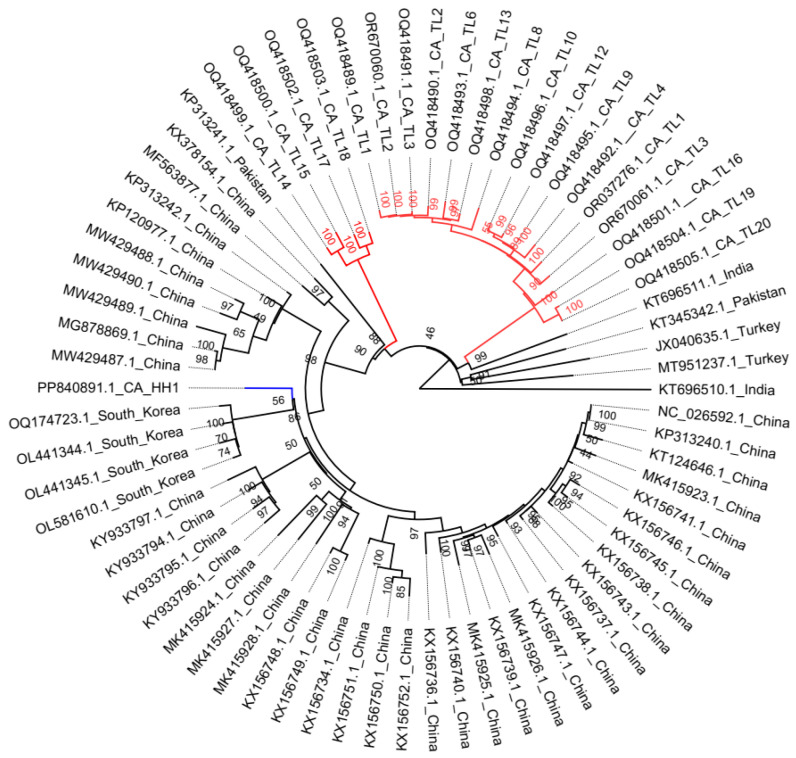
Construction of rooted maximum-likelihood phylogenetic tree upon whole genome sequences of 70 non-recombinant citrus yellow vein clearing virus (CYVCV) isolates. The clade of CYVCV California-Tulare isolates is marked with red. The clade of CYVCV California-Hacienda Heights isolate is marked with blue. Rooting method: Midpoint. Node labels display: posterior probabilities.

To ascertain the possible origin of the CYVCV California isolates, we initially assessed whether these isolates resulted from recombination. Among the 76 submitted CYVCV genome sequences, CYVCV-Kin-Del-Dec-2022 (Accession number OR251443.1), CYVCV GX-STJ (Accession number KX156742.1), CYVCV PALI (Accession number KT696512.1), CYVCV KPMI (Accession number KT696513.1), CYVCV CQ-PO (Accession number KX156735.1), and CYVCV AY221 (Accession number MW429491.1) were identified as recombinants (Supplementary Table S2). The analysis revealed no recombination events among CYVCV CA isolates. Subsequently, a maximum likelihood phylogenetic inference was performed using 70 CYVCV genome sequences, excluding the 6 identified as recombinants among the 76 submitted (Figure 3).

This analysis grouped CYVCV CA isolates into three lineages, with the CA-TL isolates forming two lineages and the CA-HH isolate forming the third. One CA-TL lineage shares the most common ancestor with an Indian CYVCV RMGI isolate (Accession number KT696511.1). In comparison, the CA-Tulare lineage containing four isolates (CYVCV-CA-TL14, 15, 17, 18) shares a common ancestor with a Pakistani CYVCV-PK isolate (KP313241.1). This supports our previous reference, suggesting that the CA-TL isolates derived from CYVCV isolates in South Asia (India and Pakistan). In contrast, the CYVCV CA-HH1 isolate shares the most recent common ancestor with the South Korean lineage. Consistent with the previous sequence alignment-based grouping data, the CA-HH1 isolate may originate from East Asia (China and South Korea). Thus, the discovery of the CYVCV CA-HH1 isolate diversifies the geographical origins of the virus in California, United States.

Additionally, this reference suggested that CYVCV likely originated from India, with the Indian CYVCV ECAI isolate (Accession number KT696510.1) connecting directly to the root of the tree. Isolates from India, Pakistan, Türkiye, and CA-TL appeared to be more closely related to the ancestor of CYVCV compared to isolates from East Asia. However, attention must be paid when interpreting phylogenetic relationships in the context of virus evolution history during a plant virus outbreak. Our data provide a look into the diverse introduction of CYVCV in California, USA. More genome sequence data of CYVCV CA-HH isolates, if available in the near future, would help yield more reliable evolutionary inferences.

It is supposed that CYVCV was probably introduced to California by unwitting homeowners, with at least two separate introductions, utilizing CYVCV-infected citrus budwoods or propagations to obtain citrus trees in their properties. Homeowners need to be better instructed about the risks of reproducing citrus trees that are not tested as pathogen-free. Sources of certified clean budwood are available at a nominal cost and will result in a sustainable and robust U.S. citrus industry.

## Data Availability

Data are contained within the article and supplementary materials. The nucleotide sequence of the complete genome sequence of CYVCV CA HH1 obtained in this study was submitted to the GenBank database under accession number PP840891.1.

## Supplementary Materials

The following supporting information can be downloaded at: https://www.mdpi.com/article/10.3390/v16091479/s1, Supplementary Table S1. The group, accession number, name, location, and collected years of 76 reported citrus yellow vein clearing virus (CYVCV) genome. Table S2. The recombination analysis of citrus yellow vein clearing virus isolates upon whole genome sequences. Six isolates were detected in over three different methods implemented in RDP4. Different algorithms abbreviation: R: RDP; G: GENECONV; B: BOOTSCAN; M: MAXCHI; C: CHIMAERA; S: SISCAN; T: 3SEQ. Supplementary Figure S1. Topographical map of California highlighting the Tulare and Hacienda Heights regions, where Citrus yellow vein clearing virus was detected. Red circles mark the locations. A legend in the corner indicates elevation ranges. (Source: Modified from https://www.california-map.org/, accessed on 14 September 2024). Figure S2. The citrus yellow vein clearing virus California isolates ingroup multiple alignment. The base-pair with frequency-based differences were marked red. Figure S3. The citrus yellow vein clearing virus (CYVCV) California isolates nucleotide diversity visualized by VIRDIC-generated heatmap. The figure incorporates intergenomic similarity values (right half) and alignment indicators (top annotation).
