# Supplementary material for: The Discovery of a Citrus Yellow Vein Clearing Virus Hacienda Heights Isolate Diversifies the Geological Origins of the Virus in California, United States
Source: Viruses. 2024 Sep 18;16(9):1479. doi: 10.3390/v16091479 (PMC11437496; doi:10.3390/v16091479)
Supplement: Supplementary file 1 [file viruses-16-01479-s001.zip › CYVCV HH Supporting Figures-Revised.pdf]

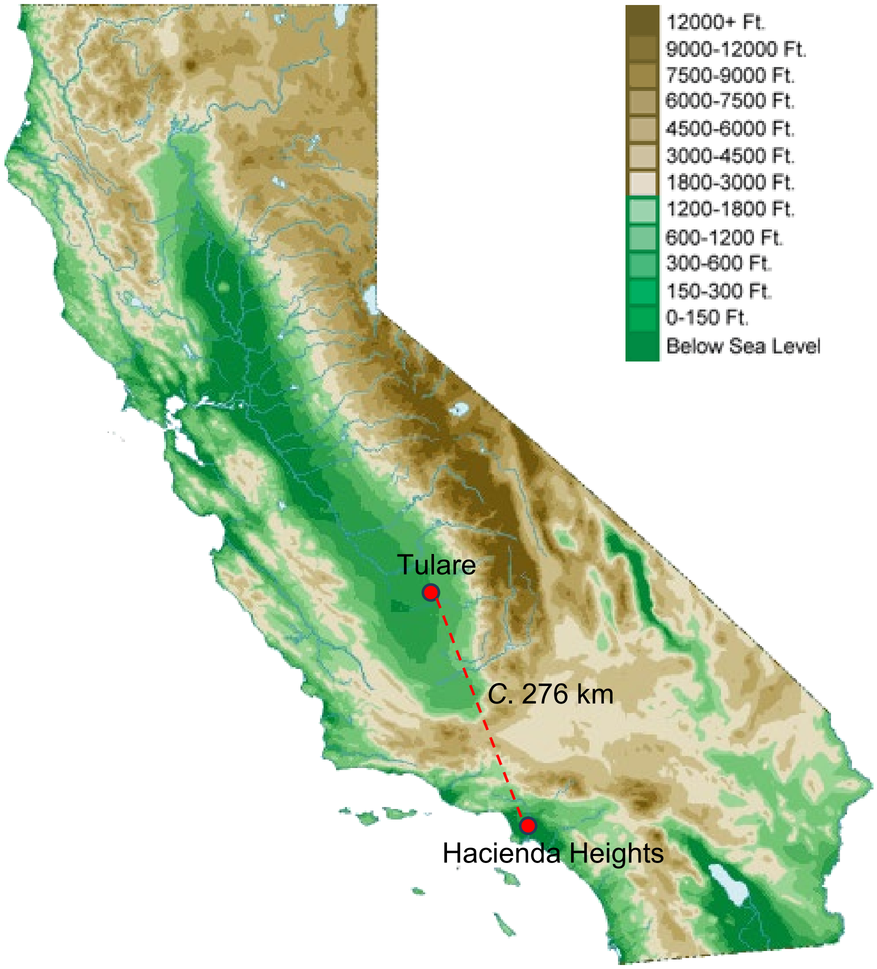

Supplementary Figure S1. Topographical map of California highlighting the Tulare and Hacienda Heights regions, where citrus yellow vein clearing virus was detected. Red circles mark the locations. A legend in the corner indicates elevation ranges. (Source: Modified fromhttps://www.california-map.org/topo-map.html)

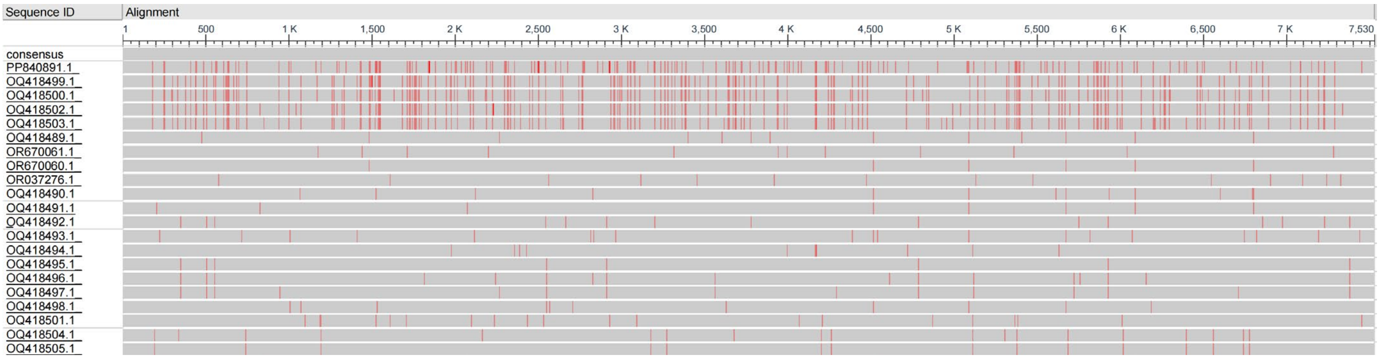

Supplementary Figure S2. The citrus yellow vein clearing virus California isolates in group multiple alignment.The base-pair with frequency-based differences were marked red.

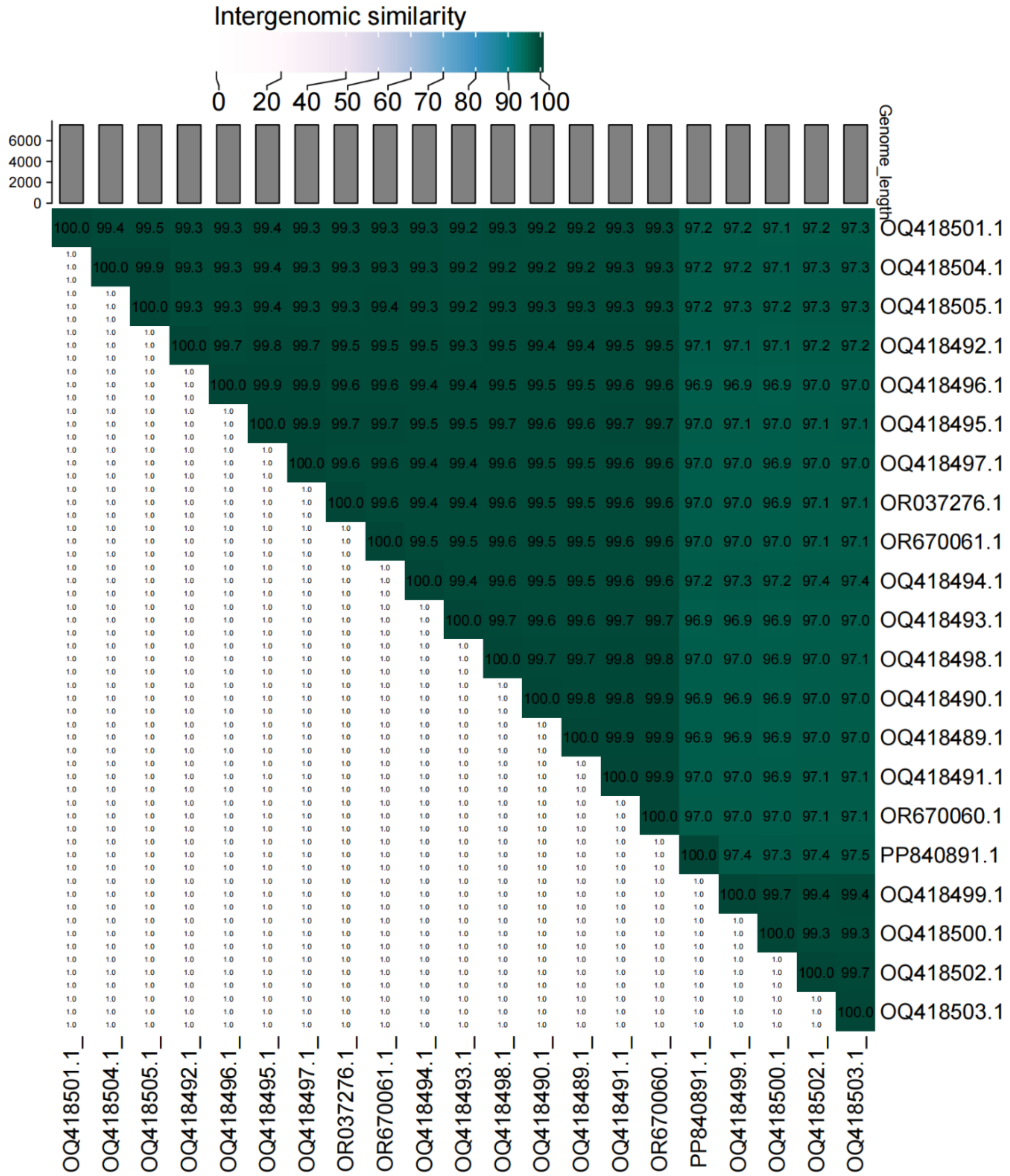

Supplementary Figure S3. The citrus yellow vein clearing virus (CYVCV) California isolates nucleotide diversity visualized by VIRDIC-generated heatmap. The figure incorporates intergenomic similarity values (right half) and alignment indicators (top annotation).
